# Changing Pre-Service Teachers’ Beliefs About Self-Regulated Learning: The Role of Refutational Texts and Instructional Analogies

**DOI:** 10.3390/bs15121623

**Published:** 2025-11-25

**Authors:** Irini Skopeliti, Natassa Kyriakopoulou, Athanasia Androutsopoulou

**Affiliations:** 1 Department of Philosophy and History of Science, National and Kapodistrian University of Athens, 15771 Athens, Greece; 2 Department of Early Childhood Education, National and Kapodistrian University of Athens, 10680 Athens, Greece; ankyriak@ecd.uoa.gr; 3 Department of Educational Science and Early Childhood Education, University of Patras, 26504 Rio, Greece; athanasia_androutsopoulou@hotmail.com

**Keywords:** SRL, refutational texts, instructional analogies, belief change

## Abstract

Pre-service teachers develop belief systems about teaching, learning, and self-regulated learning (SRL) that are often shaped by their own schooling experiences, which typically promote traditional teacher-centered views. During teacher education, they are introduced to student-centered approaches aligned with SRL, but these new beliefs often coexist with prior conceptions, resulting in internal inconsistencies. This study examined pre-service teachers’ beliefs about learning, teaching, and SRL, and evaluated whether specific instructional techniques—refutational texts and instructional analogies—could reduce conflicting beliefs. One hundred and sixty pre-service teachers completed a pretest-posttest questionnaire measuring their beliefs. Participants were randomly assigned to read one of four instructional texts about SRL-based teaching: explanatory or refutational, with or without analogies. Prior to the intervention, participants showed mixed and often conflicting beliefs about SRL. After the intervention, the refutational text combined with analogy proved most effective in reducing belief inconsistencies. These findings suggest that targeted instructional materials can support belief change by explicitly addressing and challenging prior misconceptions, thereby facilitating the adoption of coherent, student-centered perspectives aligned with SRL.

## 1. Introduction

In today’s rapidly changing world, learners are expected not only to acquire knowledge, but also to regulate their learning effectively. This ability, known as Self-Regulated Learning (SRL), involves behaviors, such as setting goals, monitoring progress, and adjusting strategies when necessary (Zimmerman, 2002). Research has shown that learners who regulate their own learning show high academic performance (Dignath & Büttner, 2008; Pintrich, 2000). SRL enhances performance because it enables learners to manage their cognitive processes, review their understanding, and adapt strategies when difficulties arise. Moreover, it comprises both skill, which refers to the metacognitive capacity to plan, monitor, and evaluate learning, and will, which refers to the motivational resources such as persistence and goal commitment that sustain engagement throughout the learning process (Pintrich, 2000). The dynamic interaction of these elements allows learners to take active control of their learning and optimize their academic outcomes.

Teacher education has a crucial role in promoting SRL. Pre-service teachers (PSTs) must learn to develop SRL skills for themselves, but they also must recognize the benefits of SRL for their future students. Knowing and appreciating the benefits of SRL, like promoting autonomy, motivation, and effective learning, is essential for PSTs to create classrooms that encourage students to self-regulate their learning. However, many PSTs enter higher education with strong beliefs about learning and teaching that have been shaped by their own school experiences (Maggioni & Parkinson, 2008). These beliefs are often in favor of traditional teacher-centered approaches and are in conflict with SRL; thus, the teacher has the absolute control for organizing and transmitting knowledge and the students cannot have control of their learning (Vosniadou et al., 2020). Because such beliefs are part of a broader belief system (Lawson et al., 2019), supporting PSTs to revise them requires more than just providing new information. The existing beliefs need to be reorganized, following the changing processes as described in the framework theory approach of conceptual change (Vosniadou, 2013).

Research in the field of conceptual change suggests that substantial changes in the students’ belief systems need to occur, and the educational tools that can support this process include refutational texts and instructional analogies. Refutational texts explicitly address common misconceptions and argue in favor of the scientifically accepted ones (Hynd, 2001). For example, in a study by Hunsu et al. (2023), refutational texts were used to address students’ misconceptions about genetic biology. The text presented a common misconception “Some people think that only higher-order organisms have deoxyribonucleic acid (DNA).” then refuted it “This is not true however;” and finally provided the correct information “DNA is the genetic material in humans and all other organisms.” (p. 4).

On the other hand, instructional analogies explain new and abstract or counterintuitive information by mapping them to familiar conceptual structures (Clement, 2013). In the study by Vosniadou and Skopeliti (2019), the counterintuitive mechanism of the day/night cycle was explained to 3rd and 5th graders in Greece using an analogy drawn from a highly familiar domain, the roasting of gyros. The text that included the analogy stated “The Earth turns around just like gyros turns around on the vertical spit while roasting. Sunlight reaches only one side of the Earth, and on this side, it is day. Similarly, the fire cooks only one side of gyros, the one that is turned toward it.” (p. 739).

Although both tools have been studied separately in the context of conceptual change and science education, their combined use has not yet been investigated. Additionally, refutational texts and instructional analogies have not been systematically used in teacher education to help PSTs develop more accurate and constructive beliefs about SRL. Addressing this gap, the present study investigated whether texts that present cognitive conflict, instructional analogies, or both can support conceptual change learning about SRL and effectively influence PSTs to adopt more positive and consistent beliefs about SRL. The finding provides valuable information for designing teacher education programs that prepare future teachers to recognize and promote the benefits of SRL in their classrooms.

## 2. Literature Review

### 2.1. SRL: Definitions and Significance

Previous research in educational psychology has shown that self-regulated learning (SRL) is one of the most influential educational models that can support the acquisition of knowledge and the development of skills necessary to meet the demands of the 21st century (Theobald, 2021; Xu et al., 2023). As Bjork et al. (2013) note, “Our complex and rapidly changing world creates a need for self-regulated and self-managed learning […]. Knowing how to manage one’s own learning activities has become, in short, an important survival tool.” (p. 418).

SRL refers to the students’ ability to plan, monitor, and regulate actively their cognition, motivation, and behavior (Zimmerman, 2002). In an educational environment, which is consistent with the principles of SRL, the students are placed at the center of the educational process, set their own learning goals, possess a range of learning strategies, select and apply the appropriate ones to regulate their learning, and adjust their behavior accordingly to meet the goals they have set (Boekaerts et al., 2000). Ιn contrast, traditional teacher-centered educational models typically consider the teacher as the main authority responsible for organizing, delivering, and assessing knowledge, while students have a more passive role as recipients of information. Such approaches may limit students’ opportunities to develop autonomy, reflection, and control over their learning (Pintrich, 2000). However, SRL does not necessarily replace teacher-centered approaches. Rather, effective instruction, based on the principles of SRL, often involves a balance between the teacher’s guidance and the learner’s autonomy. When teacher-centered elements are flexible and supportive, rather than rigid and transmissive, they can provide essential scaffolding that helps students gradually develop self-regulation (Dignath & Veenman, 2021).

Several models have been proposed for conceptualizing SRL. Zimmerman’s cyclical model describes SRL as a dynamic process that goes through three recurring phases—forethought, performance, and self-reflection—through which learners plan, implement, and evaluate their learning (Zimmerman, 2002). Pintrich (2000) emphasizes that SRL involves a complex interaction between cognition, motivation, and behaviors. Demetriou (2000) further highlights the role of cognitive and personality development in learners’ self-regulation. Efklides (2011)’s model extended to include emotional and motivational components, to emphasize the dynamic interaction between metacognitive processes and emotion. However, despite their differences, the models converge on the idea that SRL is more than just the application of learning strategies, but rather a self-directed and adaptive process, which is shaped by beliefs, prior knowledge, and contextual factors.

Empirical evidence has shown that teaching students how to regulate their learning has a positive impact on their academic performance (Dignath & Büttner, 2008; Pintrich, 2000; Zimmerman, 2008). Moreover, in higher education, SRL is particularly significant for teacher training, since trainee teachers must not only be able to regulate their own learning, but also support the development of self-regulation in their future students (Boekaerts, 1997; Zimmerman, 2002).

### 2.2. In-Service and Pre-Service Teachers’ Beliefs About Learning, Teaching and SRL

In-service and pre-service teachers’ beliefs about learning and teaching are key determinants not only of their own personal learning but also of their students’ learning, since they shape the teaching practices they adopt or plan to implement in the classroom (Buehl & Beck, 2015; Dignath & Van der Werh, 2012; Kyriakopoulou & Skopeliti, 2024; Vosniadou et al., 2023). Teachers who understand and appreciate the benefits of SRL, such as promoting autonomy, motivation, engagement, and effective learning, are more likely to create classroom environments and design teaching practices that encourage students to self-regulate their learning and emphasize active and self-directed construction of knowledge.

Despite the well-documented benefits of SRL, several studies have shown that both in-service and pre-service teachers often hold limited or fragmented understandings of its underlying processes (Michalsky, 2021; Spruce & Bol, 2015). Teachers may recognize the importance of fostering students’ autonomy and self-monitoring, but struggle to translate these principles into classroom practices (Kyriakopoulou & Skopeliti, 2024). Common challenges that teachers face are difficulties in identifying opportunities for explicit SRL instruction, balancing the teacher’s guidance with students’ independence, and reconciling traditional views of teaching, which are focused on knowledge transmission (Vosniadou et al., 2023).

These challenges are particularly relevant for pre-service teachers who are in the process of developing their professional identity and pedagogical beliefs. Thus, while this argument applies to both in-service and pre-service teachers, the present study focuses on pre-service teachers’ (PSTs) beliefs about learning and teaching, discusses how they are formed, and examines how they may change. When PSTs enter higher education to study pedagogy and teaching methods, they are not “blank sheets” on which teacher educators can simply imprint current views about effective teaching. They enter teacher education programs with well-established beliefs about the nature of knowledge, learning, and the role of the teacher in the classroom, influenced by their own previous experiences as students (Maggioni & Parkinson, 2008). Some of these beliefs are consistent with the idea that learning is a quick and innate process supported by the transmission of knowledge and well-structured activities designed by the teacher (Spruce & Bol, 2015; Vosniadou et al., 2020, 2021). Such beliefs reinforce the design of traditional teacher-centered instructional practices, in which the teacher is the only one responsible for the content, the structure, and the pace of instruction, and the main goal of instruction is knowledge transmission (Muis & Duffy, 2013). At the same time, traditional teacher-centered practices hinder the approaches that promote students’ autonomy and self-regulation (Buehl & Beck, 2015). In contrast, PSTs who acknowledge the importance of SRL are more likely to encourage goal setting, self-monitoring, reflection, and adaptive learning strategies in the classroom (Perry et al., 2008) and to develop constructive, student-centered learning environments (Minor et al., 2002).

Μany studies have shown that PSTs may express positive stances toward SRL when completing self-report questionnaires (e.g., Endedijk et al., 2013; Lombaerts et al., 2009; Michalsky, 2014), while in actual educational contexts, they often provide no or only minimal explicit instruction in self-regulation strategies to promote SRL (Bolhuis & Voeten, 2004; Dignath-van Ewijk, 2016; Dignath & Veenman, 2021; Kistner et al., 2010; Spruce & Bol, 2015). This discrepancy reflects the complex and often incoherent belief system about learning and teaching that PSTs may hold; a system that may include beliefs both consistent and inconsistent with the principles of SRL (Lawson et al., 2019; Lombaerts et al., 2009).

In order to explain this gap between theory and practice, it is often argued that beliefs about learning and teaching form a belief system that encompasses personal and intuitive perceptions; ideas that are either coherent, loosely connected, or even contradictory (Lawson et al., 2019). To investigate this hypothesis, Vosniadou et al. (2020) examined PSTs’ beliefs about learning, teaching, and SRL using the Beliefs About Learning and Teaching (BALT) questionnaire (Darmawan et al., 2020). The instrument identifies both positive and negative stances toward SRL. The results indicated that PSTs often hold intuitive beliefs that derive from personal experiences as students, which may be against the principles of SRL. After systematic exposure to constructive perspectives on learning, teaching, and SRL, PSTs started to revise their initial beliefs (Kyriakopoulou & Skopeliti, 2024; Vosniadou et al., 2020).

Vosniadou and colleagues (2020) used the framework theory approach of conceptual change to explain these findings. The framework theory was originally developed to interpret findings from science and mathematics education (Vosniadou, 2013; Vosniadou & Skopeliti, 2014). According to this approach, young students enter school having already constructed well-established beliefs about how the world works, which form a relatively coherent belief system; a theoretical framework. Since these beliefs are based on everyday experience and intuitive information, they are often resistant to change when the new scientific information is counterintuitive and/or comes in conflict with their everyday experiences. Thus, changing these initial beliefs with more sophisticated scientific ones is not an easy or immediate process. Rather, it is a long, time-consuming process, that requires many conceptual changes, ontological, epistemological, and representational ones. During this process, conflicting beliefs may coexist, sometimes giving rise to misconceptions. This view aligns with other theoretical accounts of conceptual change proposed by Carey (2009) and Chi (2013), emphasizing that learning complex or counterintuitive information requires ontological and epistemological restructuring rather than simple addition of the new information to the already established beliefs. Moreover, Rosch (1978)’s prototype theory highlights that learners’ intuitive categorizations influence the way they interpret new information and may lead to the creation of misconceptions.

From this perspective, PSTs may also construct initial beliefs about learning and teaching based on their own experiences as students. A lack of exposure to constructive teaching practices and to SRL, makes the change in PSTs’ initial beliefs particularly difficult. It is not simply a matter of providing new information, since many conceptual changes—ontological, epistemological, representational—need to take place. Instead, carefully designed interventions are required that challenge the existing well-established ideas, create cognitive conflict, and scaffold the integration of new beliefs (Sinatra & Pintrich, 2003). Among the techniques found to be effective are refutational texts, which directly address misconceptions, and instructional analogies, which facilitate the comprehension of new ideas, for which learners may lack prior experience.

### 2.3. Refutational Texts to Teach SRL

Research in the field of conceptual change has shown that students’ initial intuitive conceptions are often resistant to change, even when confronted with new evidence (Chi, 2013; Vosniadou, 2013). One instructional technique designed to address this challenge is refutational texts, which are texts that explicitly present a common misconception, usually held by students, refute it, and then present the scientifically accepted explanation (Hynd, 2001). In this way, refutational texts create a cognitive conflict, which is considered fundamental for conceptual change, and provide students with a coherent alternative conception that can replace the previous naïve one (Chow & Treagust, 2013; Madu & Orji, 2015; Vosniadou, 2007).

Research findings and meta-analyses have shown that the refutational texts are usually more effective than expository texts in promoting conceptual change learning (Christou & Prokopou, 2020; Mason et al., 2020; McCrudden & Kendeou, 2014; Tippett, 2010). This is the case because they support the process of reorganizing knowledge by prompting students to identify the conflict between their ideas and the new scientific information and by presenting counterarguments in a clear, understandable, and persuasive way (Hynd, 2001).

In the context of teacher education, refutational texts could be particularly valuable. Refutational texts can help PSTs not only recognize the limitations of naïve beliefs that support traditional teacher-centered models but also understand the benefits of student-centered models and SRL, giving them opportunities for conceptual change.

### 2.4. Instructional Analogies to Teach SRL

Another powerful tool for supporting conceptual change is instructional analogies. Instructional analogies can help students understand unfamiliar concepts by connecting them to familiar knowledge structures (Clement, 2013). By mapping relationships between a familiar domain (“base” domain) and an unfamiliar one (“target” domain), instructional analogies can scaffold students’ understanding of new information and reorganize their prior knowledge (Braasch & Goldman, 2010; Glynn, 2008). Effective use of instructional analogies can reduce cognitive load and can help students easily understand and more accurately abstract counterintuitive scientific ideas, to which they have no direct access or experience (Clement, 2013; Richland & Simms, 2015; Vamvakoussi, 2019; Vosniadou & Skopeliti, 2019).

In the context of teacher education, instructional analogies can be used to explain to the PSTs why SRL is necessary for effective learning. For example, SRL can be compared to “navigating with a map”; the learner sets the destination (learning goal), monitors the route (self-monitor), and adjusts it when obstacles arise (strategy adaptation). Such analogies may help PSTs conceptualize the abstract aspects of SRL that lie outside their previous experience, making them easier to understand and apply. Thus, using instructional analogies as an educational tool, teacher educators can explain SRL more clearly, and, at the same time, promote conceptual change.

### 2.5. Teaching About SRL to PSTs Using Both Refutational Texts and Instructional Analogies

Based on the above-reviewed literature, PSTs hold beliefs about learning, teaching, and SRL, which are grounded in personal experiences and are part of a belief system (Vosniadou et al., 2020). Changing these beliefs requires well-designed interventions that promote conceptual change. Refutational texts and instructional analogies may be effective tools because they can complement each other; refutational texts promote change by explicitly addressing misconceptions and provoking cognitive conflict, while analogies facilitate the understanding of new, complex, and abstract ideas by connecting them to familiar conceptual structures. Research findings have shown that the combination of multiple teaching techniques may be more effective in supporting conceptual change learning (Çepni & Şahin, 2012; Özmen & Naseriazar, 2018; Türk & Çalik, 2008). Therefore, the aforementioned tools could be used in combination for teaching training, providing to PSTs a dual pathway for conceptual change and helping them reorganize their initial beliefs about learning and teaching into more coherent belief systems.

### 2.6. The Present Study

The aim of the present study was to examine whether instructional texts that were refutational (thus incorporating a cognitive conflict) or were explanatory, non-refutational, and that either presented or did not present instructional analogies, can effectively influence PSTs’ beliefs about SRL. Based on the framework theory of conceptual change and using well-designed instruments, such as the BALT questionnaire (Darmawan et al., 2020; Vosniadou et al., 2020), we aimed to investigate whether different types of scientific texts could facilitate PSTs to adopt more positive and constructivist views about SRL that incorporate fewer misconceptions and greater internal consistency.

We hypothesized that PSTs’ answers in the BALT questionnaire, before reading any instructional text about the principles and the advantages of SRL, would reflect both consistencies and inconsistencies with the constructivist, student-centered approach of SRL, as reported in previous studies (Kyriakopoulou & Skopeliti, 2024; Vosniadou et al., 2020). We further expected that after reading the texts, the PSTs would show an overall improvement in their responses in the BALT questionnaire, providing a greater number of answers consistent with the principles of SRL. This improvement was anticipated across all texts, since all of them emphasized the benefits of SRL.

At the same time, we expected differential effects depending on the type of text. More specifically, we hypothesized that PSTs who read refutational texts would show more positive changes in their beliefs about SRL compared to those who read the non-refutational texts. Similarly, we expected that PSTs who read texts with instructional analogies would show more positive changes in their beliefs about SRL compared to those who read the texts without instructional analogies. The text that combined the two factors would have the strongest positive effect on PSTs’ beliefs about SRL compared to all the other texts.

To assess these changes, we used two measures: (1) the number of responses aligned with the SRL model and (2) the decrease in inconsistent responses that coexisted with SRL consistent responses. Thus, we expected that the use of refutational texts and instructional analogies would not only increase the number of responses aligned with SRL but would also reduce inconsistencies in PSTs’ belief systems, in accordance with the framework theory of conceptual change.

## 3. Methodology

### 3.1. Study Design

The present study followed a three-phase study design. In the first phase, all participants completed the BALT questionnaire, which was designed to assess their initial beliefs about SRL and served as a pretest. In the second phase, each participant read an instructional text providing information about SRL. Upon completing reading, the third phase began, during which the participants once again completed the BALT questionnaire on SRL as a posttest.

### 3.2. Participants

The study involved a convenience sample of 160 PSTs. They were all studying in a department of early childhood education at a Greek University, either the National and Kapodistrian University of Athens or the University of Patras. The majority (105 PSTs) were 18–20 years old, and almost all were females (only four males participated in the study). They were enrolled across all years of study, although most were in their first year. Their demographic details are presented in Table 1.

### 3.3. Materials

#### 3.3.1. Questionnaire

The questionnaire used in this study was the shortened version of the BALT Questionnaire (Darmawan et al., 2020; Vosniadou et al., 2020). The original extended version consisted of 65 statements/items, which addressed beliefs about learning and self-regulated learning and teaching, whereas the shorten version included only 31 statements/items. Developed by a research team at Flinders University, Australia (Vosniadou et al., 2019), the BALT questionnaire offers a methodological innovation in that it identifies not only beliefs that are consistent with SRL but also those that are inconsistent with its principles.

The items were grouped into six thematic categories. Three categories included items consistent with the principles of SRL, assessing beliefs in the benefits of SRL (SRL-Ach), constructive learning (Con-L), and constructive teaching (Con-T). The remaining three categories contained items inconsistent with the principles of SRL, assessing beliefs that deny the benefits from SRL (SRL-Inc), assume learning is innate and occurs quickly (Nat-L), and view teaching mainly as the transmission of information (Trans-T). All items of the BALT questionnaire were rated using a 6-point Likert scale (1 = strongly disagree to 6 = strongly agree), indicating the PSTs’ level of agreement with each item, and each participant was assigned a corresponding score from 1 to 6 for each item.

The 31 items of the shortened BALT questionnaire were translated into Greek by the authors, then back-translated into English and validated by the original research team. The reliability analysis of the translated questionnaire was evaluated using the Cronbach’s Alpha statistical criterion. The results showed that two items reflecting the factor of Nat-L had low factor loading, and thus, they were removed from the analysis. The two items were “Children know all they need to know about learning when they are born” (l = 0.312) and “You cannot be taught how to learn” (l = 380). After removing items with low factor loadings, the Cronbach’ Alpha criterion showed high internal consistency across all items (a = 0.859) as well as across each category separately (see Table 2).

#### 3.3.2. Texts

For the purposes of the study, four different instructional texts were developed. The first one was a simple explanatory text that provided basic information about the benefits of SRL. The explanatory text included 512 words and was the basis for the other three texts.

Excerpt from explanatory text. “Teaching is particularly effective when it incorporates the instruction of learning and self-regulation strategies. At the heart of this approach is the belief that all students can learn “how to learn”. All students can become aware of what they already know and what they still need to learn, reflect on the strategies they can use, and manage their time in ways that help them meet their goals.”

The second text was an 858-word-long refutational text that presented information about SRL in direct contrast to traditional teaching. It highlighted the benefits of the SRL model, like the explanatory text did, but also pointed out the limitations of transmissive, teacher-centered instruction.

Excerpt from refutational text. “In contrast, in a traditional educational environment, the student is treated as a passive entity, and the emphasis is on information transmission and imitation of mechanical skills—at the expense of deep understanding, which could be supported through the use of learning strategies.”

The third text was the same simple explanatory text, to which an instructional analogy was added. The analogy was designed to illustrate the principles of the SRL, using a familiar situation; that of a tourist visiting a foreign country for the first time. The analogy text was 637 words long.

Excerpt from explanatory with analogy text. “In a learning environment that aims to teach SRL strategies, the student is like a tourist visiting a new place for the first time, who chooses to explore it alone. Just as the tourist decides on his/her own which sights s/he want to visit, how to get there, what information s/he want to receive about them, and how much time to spend at each one, the same applies to the students: they decide what more they need to learn in order to fully understand something, where to search for the necessary information, which information to keep from what they found, and how much time to spend until they feel they have understood something.”

The fourth text combined the refutational structure and the content of the instructional analogy, comprising 1080 words. Based on the simple explanatory text, it presented the advantages of teaching based on the principles of SRL, adding the contrast to traditional teacher-centered teaching, while at the same time incorporating the tourist analogy to illustrate the principles of SRL. It aimed to enhance understanding by emphasizing both the shortcomings of transmissive teaching and the autonomy and strategic decision-making involved in self-regulated learning.

Excerpt from refutational with analogy text. “In contrast, in a traditional learning environment, the student is like a tourist visiting a place with the guidance of a tour guide. In this case, the tourist sees the sights chosen by the guide, receives only the information provided by the guide, and spends time at each place based on how much the guide deems sufficient. Similarly, the student in a traditional learning environment focuses on school subjects selected by the teacher, receives only the information the teacher chooses to present, and engages with that information for as long as the teacher considers appropriate.”

### 3.4. Procedure

Participants were informed about the study’s purpose and content and received clarifications and explanations if needed. They then provided written informed consent to participate. The entire study was conducted online via a Google Forms link, which was shared with those who agreed to participate. Initially, they completed the BALT questionnaire (pretest). Immediately after that, while remaining on the same link, they were randomly assigned one of the four texts to read. No time limit was imposed for reading the text so as to avoid placing any pressure on the participants. Thus, each participant was allowed to spend as much time as needed to comprehend the text information. Upon completing the reading, participants immediately responded to the BALT questionnaire again (posttest) through the same form. This procedure allowed for each PST’s pretest and posttest responses, along with the specific text read, to be recorded without collecting any personal information.

## 4. Results

Participants’ responses were evaluated both individually and according to each thematic category, namely: Achievements from SRL (SRL-Ach), benefits from Constructive Teaching (Con-T), benefits from Constructive Learning (Con-L), SRL Inconsistency (SRL-Inc), Learning as a Natural process (Nat-L), and Teaching as Transmission of information (Trans-T). For the analysis of the results, the scores of certain items—specifically those assessing beliefs inconsistent with the principles of SRL—were reversed. Thus, statements like “Learning how to use learning strategies is a waste of time” (SRL-Inc), “It is a waste of time to try to understand something that does not make sense to you the first time you read it” (Nat-L), and “The main goal of teaching is to increase the amount of knowledge in the students’ memory” (Trans-T) were scored in reverse, because sophisticated beliefs about SRL are associated with rejecting such views.

### 4.1. Pretest vs. Posttest Performance

#### 4.1.1. Overall Performance

The participants’ average scores across all items were calculated, resulting in a mean score for each participant in both the pretest and the posttest. These mean scores were subjected to a non-parametric Wilcoxon analysis, and the results showed that each text individually led to improved responses from pretest to posttest. According to the statistical test, the improvement associated with all texts and with each text separately was statistically significant (see Table 3).

#### 4.1.2. Performance in Each Belief Category

The participants’ average scores were calculated for each belief category separately, resulting in six mean scores for each participant in the pretest and posttest. All texts and each text separately improved PSTs’ beliefs from pretest to posttest in the category of beliefs supporting the benefits of SRL. Τhe non-parametric statistical analysis showed that this improvement was significant for all texts as a whole and for the Explanatory with and without analogy texts separately (see Table 4), while for the refutational texts the observed differences were not statistically significant, as indicated in the table by n.s. (not significant).

In the category of beliefs supporting constructive teaching, it was found that all texts, as well as each text individually, improved PSTs’ beliefs from pretest to posttest. However, the non-parametric statistical analysis showed that this improvement was not significant in any case. It appears that participants already knew and recognized the benefits of constructive teaching from the outset, as indicated by their high pretest scores. Thus, although there was some improvement in the posttest, there was limited space for further improvement (see Table 5).

The same findings were observed in the category of beliefs supporting Constructive learning. There was an improvement in PSTs’ performance from pretest to posttest, but it was very small, since most of the participants were assessed positively from the outset in terms of the principles of constructive learning (see Table 6).

In the category of beliefs that suggested SRL does not lead to academic benefits, an improvement in the PSTs’ performance was observed, since more of the participants expressed disagreement with the statements in the posttest compared to the pretest. However, this improvement was small, and the non-parametric analysis showed that it was statistically significant only when examining all texts combined, but not for each text individually (see Table 7).

The same findings were observed in the category of statements which suggested that learning is innate and occurs quickly. The participants’ performance improved, since more of them expressed disagreement with the statements in the posttest compared to the pretest. However, this improvement was small, and the non-parametric analysis showed that it was not statistically significant. In the category of Nat-L, the PSTs showed high performance from the beginning and, as a result, there was limited space for further improvement (see Table 8).

Finally, in the category of statements supporting that teaching is mainly the transmission of information from teacher to student, all texts together and each text individually improved PSTs’ responses from pretest to posttest. In the pretest, the participants’ scores were relatively low across all conditions, whereas after the interventions, the posttest scores increased significantly. Τhe non-parametric statistical analysis showed that this improvement was significant for all texts in total and for each text individually as shown in Table 9. A greater improvement was found in the case of the refutational text with analogy; the mean score in the pretest was 3.209, and 4.101 in the posttest. The improvement in the other texts was below 0.650 points.

Based on the above, it appears that all texts contributed to improving participants’ understanding of SRL and its principles. However, the most notable change was observed in the category of statements supporting the idea that teaching is limited to the transmission of knowledge from teacher to student, where participants’ pretest scores were relatively low, allowing substantial improvement. Following the interventions, posttest scores improved significantly according to the non-parametric analysis presented above. When examining the effects of each text individually, we noticed significant gains for all conditions, while the largest improvement in the mean scores was observed following the refutational text with analogy, suggesting that the combination of refutational text with instructional analogies may have the strongest impact on changing participants’ beliefs in this category. However, in most other categories (Con-T, Con-L, and Nat-L), participants seemed to already have a solid understanding from the beginning, leaving little room for improvement. In the cases of SRL-Ach and SRL-Inc, a small improvement was found, mostly when analyzing the results coming from all texts combined.

### 4.2. Coexistence of Consistent and Inconsistent Beliefs in Pretest and Posttest

In order to examine the hypothesis of coexistence, composite scores were computed by averaging responses across all items within each belief category. As shown in Table 10, both consistent and conflicting beliefs related to SRL theories co-occurred in participants’ responses across all text groups in the pretest. More specifically, a high level of co-existence was observed between Transmissive Teaching and (a) Constructive Learning, (b) Constructive Teaching, and (c) SRL-Achievement in the pretest. Although PSTs agreed with beliefs that viewed learning as a constructive process, supported by constructive teaching practices, and the instruction of learning strategies, they simultaneously supported beliefs emphasizing that the main goal of teaching is to increase the amount of knowledge in the students’ memory. This pattern was not observed between Natural Learning and SRL-Inconsistency and the positive beliefs about SRL (which is why the specific results are not reported in Table 10).

The same analysis was conducted in the posttest. A decrease in the co-existence of conflicting beliefs was observed across all text conditions. The reduction was most substantial following the refutational text with analogy (below 20%). The detailed results are presented in Table 11.

Based on these findings, it appears that while all texts reduced inconsistencies in the PSTs’ responses in the posttest, the refutational with analogy text achieved a greater reduction, indicating that the combination of the two teaching techniques had the strongest impact.

## 5. Discussion

In the present study, we aimed to investigate PSTs’ initial beliefs about SRL and examine whether well-designed instructional texts could shift initial beliefs toward perspectives consistent with contemporary learning theories, thereby fostering a deeper understanding of the benefits of SRL. Using the BALT questionnaire (Darmawan et al., 2020; Vosniadou et al., 2020), we found that beliefs consistent with SRL often coexisted with inconsistent beliefs, indicating that the construction of beliefs about learning, teaching, and SRL is best understood as a conceptual change learning process. To address this, we designed four instructional texts, varying in type (explanatory or refutational) and in the presence or absence of instructional analogies. The combination of refutational type with analogies was particularly effective in improving participants’ responses in the posttest and in reducing internal consistency compared to the other texts. In what follows, we discuss these results in detail and in relation to our hypotheses and examine their implications.

### 5.1. PSTs’ Belief System About Teaching, Learning, and SRL: Evidence of Conceptual Change

According to our first hypothesis, the BALT questionnaire would reveal that PSTs hold beliefs that are consistent and inconsistent with SRL in the pretest. The findings confirmed this prediction, showing that the BALT questionnaire had very good applicability to the Greek context, revealing a similar pattern of responses to that reported in previous studies, with beliefs consistent with SRL coexisting with inconsistent ones (Darmawan et al., 2020; Lawson et al., 2019; Vosniadou et al., 2020). More specifically, in the positive belief categories (ConL, ConT, and SRL-Ach), Greek PSTs performed almost identically to participants in earlier research, indicating that they recognize the benefits of SRL. Interestingly, their performance in the negative belief categories (SRL-Inc and NatL) was relatively high, while their performance in the Transmissive Teaching category remained particularly low. These results indicate that, although PSTs acknowledge the value of SRL, and partially reject simplistic negative conceptions, they continue to maintain transmissive views about teaching. Such coexistence provides evidence of conceptual change, where new understanding develops alongside previously held conceptions, highlighting the complex and sometimes contradictory nature of the PSTs’ belief systems (Kyriakopoulou & Skopeliti, 2024; Vosniadou et al., 2020).

The gap between theory and practice, already described in the literature (Bolhuis & Voeten, 2004; Dignath-van Ewijk, 2016; Dignath & Veenman, 2021; Kistner et al., 2010; Spruce & Bol, 2015), can be attributed to the coexistence of beliefs consistent to SRL with inconsistent ones. Addressing these inconsistencies is essential; PSTs must move from conflicting belief systems toward more coherent ones, aligned with SRL principles, in order to support their implementation in their future classrooms. This transformation requires several conceptual changes to resolve internal inconsistencies within the PSTs’ belief systems (Kyriakopoulou & Skopeliti, 2024).

### 5.2. Need for Targeted Interventions: The Effects of Instructional Texts on PSTs’ Beliefs

According to the framework theory of conceptual change, this coexistence of contradictory beliefs highlights the need for well-designed interventions (Vosniadou, 2007). The simple presentation of sophisticated beliefs is insufficient. This may explain why the simple explanatory instructional text used in the present study improved PSTs’ beliefs about SRL, but did not resolve the internal inconsistencies. The finding is consistent with previous research highlighting that teaching techniques—such as refutational texts and instructional analogies—can facilitate the integration of more sophisticated and coherent beliefs (Clement, 2013; Tippett, 2010).

#### 5.2.1. Effects of Each Instructional Technique Individually

When applied separately, each instructional technique produced only partial benefits. Both the refutational text without the analogy and the explanatory text with the analogy increased the positive PSTs’ beliefs about the SRL, but internal inconsistencies remained. In these cases, the beliefs about the positive effects of transmissive teaching continued to exist in their posttest responses, together with their positive beliefs about the SRL. This finding is in line with previous studies that highlight the benefits of combining different teaching techniques (Çepni & Şahin, 2012; Özmen & Naseriazar, 2018; Türk & Çalik, 2008).

#### 5.2.2. The Power of Techniques When Combined

The combination of the two instructional techniques proved particularly effective. The refutational with analogy text not only increased the number of responses consistent with SRL but also reduced the number of responses consistent with transmissive teaching, thereby reducing internal inconsistencies in the PSTs compared to the other texts. Therefore, the combination of explicitly challenging naïve beliefs through cognitive conflict with scaffolding new abstract ideas via familiar representations can promote conceptual change learning and support complete understanding of abstract concepts.

### 5.3. Implications for Teacher Education

The present study contributes to the growing body of research on teacher education and the instruction of SRL in the classroom. Previous research findings have shown that PSTs’ belief systems influenced their instructional practices (Dignath & Van der Werh, 2012; Kyriakopoulou & Skopeliti, 2024; Vosniadou et al., 2023), highlighting the need to support PSTs in understanding and implementing SRL, given its critical role in promoting lifelong learning and academic success (Bjork et al., 2013; Dignath & Büttner, 2008; Zimmerman, 2008). While existing training programs emphasize how to foster SRL in the classroom (Dignath & Büttner, 2008; Dignath & Van der Werh, 2012; Harding et al., 2017; Kistner et al., 2010; Perry et al., 2015; Pintrich, 2000; Zimmerman, 2002), our findings suggest that this is not enough.

Teacher education should first identify and challenge PSTs’ beliefs about learning and teaching. These beliefs are usually created from PSTs’ own schooling (Maggioni & Parkinson, 2008). Once such beliefs become established, they are resistant to change and may hinder the understanding of SRL (Vosniadou et al., 2020). It is therefore crucial to intervene before these belief systems become entrenched (Dignath & Van der Werh, 2012).

Well-designed instructional approaches that promote conceptual change can serve as powerful tools in this effort. Our findings show that combining refutational texts with instructional analogies can effectively address this issue by promoting conceptual change and helping PSTs develop more coherent, constructivist-oriented belief systems. This underscores the importance of embedding such approaches in teacher education curricula, so that future teachers are not only theoretically aware of SRL and its benefits, but also able to overcome the doubts and misconceptions rooted in their own experiences as students, which often create persistent obstacles to the full understanding and acceptance of new information.

### 5.4. Limitations of the Study

The present study, however, is subject to some limitations. The sample size was relatively small and convenient, since it was drawn from two teacher education university departments in Greece. This limits the generalizability of the findings to the broader population of PSTs. Future research would benefit from replicating the study with a larger and more diverse sample, including participants from different universities and cultural contexts, in order to examine whether the observed effects can be confirmed across different contexts.

An additional limitation is that the impact of the teaching strategy—refutational text with instructional analogy—was examined only in the short-term, since the posttest was administered immediately after reading the text. A follow-up measurement would be useful to assess the potential long-term effects of the intervention.

Another limitation concerns the scope of the measured outcomes. While the study showed that refutational texts combined with instructional analogies influenced PSTs’ beliefs about SRL, it remains unclear whether these changes translate into changes in classroom practices. Future research should investigate the extent to which conceptual change in the PSTs’ beliefs is followed by changes in their instructional behavior.

## 6. Conclusions

In conclusion, while the present study has certain limitations, its findings make a meaningful contribution by showing that carefully designed texts—particularly those combining refutation and analogy—can effectively foster conceptual change in PSTs’ beliefs about SRL and its benefits. This highlights a promising direction for teacher education, offering both theoretical and practical insights into how future teachers can be better prepared to understand and consequently promote SRL in their classroom. The results underscore that interventions must not merely present SRL concepts but actively engage PSTs in challenging their naïve beliefs and integrating new ideas.

## Figures and Tables

**Table 1:** Participants’ demographics.

		Frequency	Percent
Age	18–20	105	65.6
	21–25	43	26.9
	26–31	1	0.6
	31–40	4	2.5
	Above 40	7	4.4
Gender	Male	4	2.5
	Female	156	97.5
Study year	1st	109	68.1
	2nd	11	6.9
	3rd	6	3.8
	4th	33	20.6
	Above 4th	1	0.6
University	Patras	128	80.0
	Athens	32	20.0

**Table 2:** Descriptive statistics and measurement model results for the shortened version of BALT in Greek PST population.

Name	Description	Mean	SD	Factor Loading
SRL-Ach	Cronbach’s Alpha = 0.743			
	Students need to continuously use their knowledge about learning during a lesson to develop a good understanding	4.181	1.159	0.526
	When students learn how to learn their performance improves	5.025	1.015	0.606
	When students learn detailed strategies for learning they develop better understanding	4.081	1.364	0.548
	When students can learn to self-regulate their learning, their achievement improves	4.856	1.033	0.675
	When students learn to regulate their learning in a lesson their understanding improves	4.619	0.990	0.674
Con-T	Cronbach’s Alpha = 0.798			
	It is important for teachers to teach students ways to remember new information	5.175	0.949	0.583
	An important task for teachers is to teach students strategies for learning	4.450	1.196	0.659
	It is important for teachers to teach students ways to organize new information	4.875	0.930	0.811
	Teachers should teach students ways to integrate new information with their existing knowledge	4.925	0.901	0.659
Con-L	Cronbach’s Alpha = 0.639			
	Learning involves the development of meaningful knowledge structure	5.119	0.796	0.414
	Learning is better when students connect new information to what they already know	5.306	0.832	0.437
	Learning requires organization of information in memory	4.794	0.965	0.483
	Learning is better when students can evaluate their level of understanding	4.738	1.037	0.622
	Effective learning requires the ability to detect gaps in one’s own understanding	4.844	0.975	0.678
SRL-Inc	Cronbach’s Alpha = 0.758			
	You do not need to understand the process of learning to be a good student	4.113	1.427	0.411
	Students don’t need to be able to describe their learning strategies	3.638	1.371	0.448
	Learning how to use learning strategies is a waste of time	5.325	1.096	0.699
	Using learning strategies does not result in better learning	5.106	1.019	0.703
	Being taught learning strategies explicitly does not help students learn	4.775	1.159	0.749
	Learning strategies are only needed when students meet a difficulty during learning	4.675	1.113	0.541
Nat-L	Cronbach’s Alpha = 0.743			
	If students are going to be able to learn something it will make sense to them the first time they hear it	5.744	0.712	0.408
	It is a waste of time to try to understand something that does not make sense to you the first time you read it	5.619	0.846	0.575
	Students who are smart must have been good learners	5.488	0.801	0.510
	Effective learning is always quick	5.156	1.025	0.688
	Successful students learn things quickly	5.158	1.024	0.563
Trans-T	Cronbach’s Alpha = 0.740			
	Teaching mostly involves the provision of information	2.525	1.293	0.648
	The main goal of teaching is to increase the amount of knowledge in the students’ memory	4.000	1.313	0.413
	The main task of the teacher is to dispense information	3.381	1.475	0.834
	The most important task of teachers consists of teaching subject knowledge	3.350	1.402	0.731

**Table 3:** Mean performance in pretest and posttest as a function of text-group.

	Pretest		Posttest		Mean Difference
Texts	Mean	SD	Mean	SD	Significance
Explanatory without analogy	4.620	0.476	4.735	0.563	[Z = −2.922, N = 160, *p* = 0.003]
Refutational without analogy	4.671	0.553	4.726	0.452	[Z = −1.949, N = 160, *p* = 0.045]
Explanatory with analogy	4.699	0.366	4.802	0.416	[Z = −2.037, N = 160, *p* = 0.042]
Refutational with analogy	4.658	0.538	4.749	0.639	[Z = −2.492, N = 160, *p* = 0.01]
Total	4.656	0.481	4.752	0.528	[Z = −3.781, N = 160, *p* < 0.001]

**Table 4:** Mean performance in pretest and posttest as a function of text-group in SRL-Ach.

	Pretest		Posttest		Mean Difference
Texts	Mean	SD	Mean	SD	Significance
Explanatory without analogy	4.507	0.787	4.857	0.733	[Z = −4.079, N = 160, *p* < 0.001]
Refutational without analogy	4.513	0.897	4.647	0.822	[Z = −0.609, N = 160, n.s.]
Explanatory with analogy	4.519	0.754	4.930	0.608	[Z = −3.628, N = 160, *p* < 0.001]
Refutational with analogy	4.687	0.703	4.719	0.834	[Z = −0.425, N = 160, n.s.]
Total	4.552	0.779	4.803	0.747	[Z = −4.689, N = 160, *p* < 0.001]

**Table 5:** Mean performance in pretest and posttest as a function of text-group in Con-T.

	Pretest		Posttest		Mean Difference
Texts	Mean	SD	Mean	SD	Significance
Explanatory without analogy	4.915	0.774	4.991	0.831	[Z = −0.873, N = 160, n.s.]
Refutational without analogy	4.692	0.925	4.742	0.975	[Z = −0.065, N = 160, n.s.]
Explanatory with analogy	4.824	0.743	4.946	0.852	[Z = −1.819, N = 160, n.s.]
Refutational with analogy	4.838	0.784	4.856	0.848	[Z = −0.584, N = 160, n.s.]
Total	4.818	0.784	4.883	0.867	[Z = −0.980, N = 160, n.s.]

**Table 6:** Mean performance in pretest and posttest as a function of text-group in Con-L.

	Pretest		Posttest		Mean Difference
Texts	Mean	SD	Mean	SD	Significance
Explanatory without analogy	4.882	0.625	4.932	0.831	[Z = −1.120, N = 160, n.s.]
Refutational without analogy	5.020	0.642	5.000	0.975	[Z = −0.123, N = 160, n.s.]
Explanatory with analogy	5.056	0.433	5.081	0.852	[Z = −0.123, N = 160, n.s.]
Refutational with analogy	4.973	0.655	4.989	0.848	[Z = −0.500, N = 160, n.s.]
Total	4.960	0.595	4.992	0.867	[Z = −0.399, N = 160, n.s.]

**Table 7:** Mean performance in pretest and posttest as a function of text-group in SRL-Inc.

	Pretest		Posttest		Mean Difference
Texts	Mean	SD	Mean	SD	Significance
Explanatory without analogy	4.640	0.763	4.765	0.853	[Z = −1.474, N = 160, n.s.]
Refutational without analogy	4.556	0.865	4.739	0.687	[Z = −1.603, N = 160, n.s.]
Explanatory with analogy	4.608	0.746	4.766	0.797	[Z = −1.648, N = 160, n.s.]
Refutational with analogy	4.590	0.874	4.649	0.827	[Z = −0.362, N = 160, n.s.]
Total	4.605	0.798	4.733	0.799	[Z = −2.582, N = 160, *p* = 0.01]

**Table 8:** Mean performance in pretest and posttest as a function of text-group in Nat-L.

	Pretest		Posttest		Mean Difference
Texts	Mean	SD	Mean	SD	Significance
Explanatory without analogy	5.404	0.599	5.468	0.661	[Z = −1.203, N = 160, n.s.]
Refutational without analogy	5.480	0.560	5.480	0.516	[Z = −0.142, N = 160, n.s.]
Explanatory with analogy	5.503	0.561	5.411	0.579	[Z = −1.320, N = 160, n.s.]
Refutational with analogy	5.368	0.812	5.454	0.727	[Z = −1.064, N = 160, n.s.]
Total	5.433	0.636	5.454	0.629	[Z = −0.488, N = 160, n.s.]

**Table 9:** Mean performance in pretest and posttest as a function of text-group in Trans-T.

	Pretest		Posttest		Mean Difference
Texts	Mean	SD	Mean	SD	Significance
Explanatory without analogy	3.125	0.958	3.741	0.983	[Z = −5.504, N = 160, *p* < 0.001]
Refutational without analogy	3.525	1.119	3.950	0.927	[Z = −2.655, N = 160, *p* = 0.008]
Explanatory with analogy	3.534	1.048	3.939	0.914	[Z = −3.189, N = 160, *p* = 0.001]
Refutational with analogy	3.209	1.018	4.101	0.976	[Z = −4.489, N = 160, *p* < 0.001]
Total	3.314	1.031	3.909	0.956	[Z = −8.149, N = 160, *p* < 0.001]

**Table 10:** Co-existence of beliefs consistent and inconsistent with SRL theory in pretest.

Texts	Explanatory Without Analogy (% of Total)	Refutational Without Analogy (% of Total)	Explanatory with Analogy (% of Total)	Refutational with Analogy (% of Total)
Trans-T vs. Con-L	32% Disagree to Trans-T	47% Disagree to Trans-T	57% Disagree to Trans-T	43% Disagree to Trans-T
	96% Agree to Con-L	100% Agree to Con-L	100% Agree to Con-L	97% Agree to Con-L
	66% Agree to both	53% Agree to both	43% Agree to both	54% Agree to both
Trans-T vs. Con-T	32% Disagree to Trans-T	47% Disagree to Trans-T	57% Disagree to Trans-T	43% Disagree to Trans-T
	95% Agree to Con-T	93% Agree to Con-T	95% Agree to Con-T	97% Agree to Con-T
	66% Agree to both	50% Agree to both	43% Agree to both	57% Agree to both
Trans-T vs. SRL-Ach	32% Disagree to Trans-T	47% Disagree to Trans-T	57% Disagree to Trans-T	43% Disagree to Trans-T
	88% Agree to SRL-Ach	93% Agree to SRL-Ach	89% Agree to SRL-Ach	92% Agree to SRL-Ach
	61% Agree to both	50% Agree to both	43% Agree to both	54% Agree to both

**Table 11:** Co-existence of beliefs consistent and inconsistent with SRL theory in posttest.

Texts	Explanatory Without Analogy (% of Total)	Refutational Without Analogy (% of Total)	Explanatory with Analogy (% of Total)	Refutational with Analogy (% of Total)
Trans-T vs. Con-L	57% Disagree to Trans-T	70% Disagree to Trans-T	70% Disagree to Trans-T	78% Disagree to Trans-T
	95% Agree to Con-L	100% Agree to Con-L	100% Agree to Con-L	95% Agree to Con-L
	43% Agree to both	30% Agree to both	30% of total Agree to both	16% Agree to both
Trans-T vs. Con-T	57% Disagree to Trans-T	70% Disagree to Trans-T	70% Disagree to Trans-T	78% Disagree to Trans-T
	93% Agree to Con-T	94% Agree to Con-T	95% Agree to Con-T	95% Agree to Con-T
	43% Agree to both	30% Agree to both	30% Agree to both	19% Agree to both
Trans-T vs. SRL-Ach	57% Disagree to Trans-T	70% Disagree to Trans-T	70% Disagree to Trans-T	78% Disagree to Trans-T
	98% Agree to SRL-Ach	93% Agree to SRL-Ach	100% Agree to SRL-Ach	95% Agree to SRL-Ach
	43% Agree to both	30% Agree to both	30% Agree to both	19% Agree to both

## Data Availability

Data cannot be shared openly but are available on request from the authors.

## References

[B1-behavsci-15-01623] Bjork R. A., Dunlosky J., Kornell N. (2013). Self-regulated learning: Beliefs, techniques, and illusions. Annual Review of Psychology.

[B2-behavsci-15-01623] Boekaerts M. (1997). Self-regulated learning: A new concept embraced by researchers, policy makers, educators, teachers, and students. Learning and Instruction.

[B3-behavsci-15-01623] Boekaerts M., Pintrich P. R., Zeidner M. (2000). Handbook of self-regulation.

[B4-behavsci-15-01623] Bolhuis S., Voeten M. J. M. (2004). Teachers’ conceptions of student learning and own learning. Teachers and Teaching: Theory and Practice.

[B5-behavsci-15-01623] Braasch J. L. G., Goldman S. R. (2010). The role of prior knowledge in learning from analogies in science texts. Discourse Processes.

[B6-behavsci-15-01623] Buehl M. M., Beck J. S., Fives H., Gill M. G. (2015). The relationship between teachers’ beliefs and teachers’ practices. International handbook of research on teachers’ beliefs.

[B7-behavsci-15-01623] Carey S. (2009). The origin of concepts.

[B8-behavsci-15-01623] Chi M. T., Vosniadou S. (2013). Two kinds and four sub-types of misconceived knowledge, ways to change it, and the learning outcomes. International handbook of research on conceptual change.

[B9-behavsci-15-01623] Chow T.-C. F., Treagust D. F. (2013). An intervention study using cognitive conflict to foster conceptual change. Journal of Science and Mathematics Education in Southeast Asia.

[B10-behavsci-15-01623] Christou K. P., Prokopou A. (2020). Using refutational text to address the multiplication makes bigger misconception. Educational Journal of the University of Patras UNESCO Chair.

[B11-behavsci-15-01623] Clement J. J. (2013). Roles for explanatory models and analogies in conceptual change. International handbook of research on conceptual change.

[B12-behavsci-15-01623] Çepni S., Şahin Ç. (2012). Effect of different teaching methods and techniques embedded in the 5E instructional model on students’ learning about buoyancy force. International Journal of Physics and Chemistry Education.

[B13-behavsci-15-01623] Darmawan I. G. N., Vosniadou S., Lawson M. J., Van Deur P., Wyra M. (2020). The development of an instrument to test pre-service teachers’ beliefs consistent and inconsistent with self-regulation theory. British Journal of Educational Psychology.

[B14-behavsci-15-01623] Demetriou A., Boekaerts M., Pintrich P. R., Zeidner M. (2000). Organization and development of self-understanding and self-regulation: Toward a general theory. Handbook of self-regulation.

[B15-behavsci-15-01623] Dignath C., Büttner G. (2008). Components of fostering self-regulated learning among students. A meta-analysis on intervention studies at primary and secondary school level. Metacognition and Learning.

[B16-behavsci-15-01623] Dignath C., Van der Werh G. (2012). What teachers think about self-regulated learning: Investigating teacher beliefs and teacher behavior of enhancing students’ self-regulation. Educational Research International.

[B17-behavsci-15-01623] Dignath C., Veenman M. V. J. (2021). The role of direct strategy instruction and indirect activation of self-regulated learning—Evidence from classroom observation studies. Educational Psychology Review.

[B18-behavsci-15-01623] Dignath-van Ewijk C. (2016). What determines whether teachers enhance self-regulated learning? Predicting teachers’ reported promotion of self-regulated learning by teacher beliefs, knowledge, and self-efficacy. Frontline Learning Research.

[B19-behavsci-15-01623] Efklides A. (2011). Interactions of metacognition with motivation and affect in self-regulated learning: The MASRL model. Educational Psychologist.

[B20-behavsci-15-01623] Endedijk M. D., Brekelmans M., Verloop N., Sleegers P. J. C., Vermunt J. D. (2013). Individual differences in student teachers’ self-regulated learning: An examination of regulation configurations in relation to configurations in relation to conceptions of learning to teach. Learning and Individual Differences.

[B21-behavsci-15-01623] Glynn S. M., Mikelskis-Seifert S., Ringelband U., Brückmann M. (2008). Making science concepts meaningful to students: Teaching with analogies. Four decades of research in science education: From curriculum development to quality improvement.

[B22-behavsci-15-01623] Harding S. M., Nibali N., Griffin P., Graham L., English N. (2017). Teaching self-regulated learning in victorian classrooms. Australian Association for Research in Education (AARE) Conference.

[B23-behavsci-15-01623] Hunsu N. J., Adesope O., McCrudden M. T. (2023). The effects of text structure on students’ use of comprehension strategies and cognitive outcomes during science text processing. Frontiers in Education.

[B24-behavsci-15-01623] Hynd C. (2001). Refutational texts and the change process. International Journal of Educational Research.

[B25-behavsci-15-01623] Kistner S., Rakoczy K., Otto B., Dignath-van Ewijk C., Büttner G., Klieme E. (2010). Promotion of self-regulated learning in classrooms: Investigating frequency, quality, and consequences for student performance. Metacognition and Learning.

[B26-behavsci-15-01623] Kyriakopoulou N., Skopeliti I. (2024). Pre-service teachers’ epistemic and educational beliefs about learning, teaching and students’ cognitive engagement: Their effect on selecting teaching practices. International Journal of Early Childhood Education.

[B27-behavsci-15-01623] Lawson M. J., Vosniadou S., Van Deur P., Wyra M., Jeffries D. (2019). Teachers’ and students’ belief systems about the self-regulation of learning. Educational Psychology Review.

[B28-behavsci-15-01623] Lombaerts K., De Backer F., Engels N., Van Braak J., Athanasou J. (2009). Development of the self-regulated learning teacher belief scale. European Journal of Psychology of Education.

[B29-behavsci-15-01623] Madu B. C., Orji E. (2015). Effects of cognitive conflict instructional strategy on students’ conceptual change in temperature and heat. SAGE Open.

[B30-behavsci-15-01623] Maggioni L., Parkinson M. M. (2008). The role of teacher epistemic cognition, epistemic beliefs, and calibration in instruction. Educational Psychology Review.

[B31-behavsci-15-01623] Mason L., Borella E., Diakidoy I. A. N., Butterfuss R., Kendeou P., Carretti B. (2020). Learning from refutation and standard expository science texts: The contribution of inhibitory functions in relation to text type. Discourse Processes.

[B32-behavsci-15-01623] McCrudden M. T., Kendeou P. (2014). Exploring the link between cognitive processes and learning from refutational text. Journal of Research in Reading.

[B33-behavsci-15-01623] Michalsky T. (2014). Developing the SRL-PV assessment scheme: Preservice teachers’ professional vision for teaching self-regulated learning. Studies in Educational Evaluation.

[B34-behavsci-15-01623] Michalsky T. (2021). Preservice and inservice teachers’ noticing of explicit instruction for self-regulated learning strategies. Frontiers in Psychology.

[B35-behavsci-15-01623] Minor L. C., Onwuegbuzie A. J., Witcher A. E., James T. L. (2002). Preservice teachers’ educational beliefs and their perceptions of characteristics of effective teachers. The Journal of Educational Research.

[B36-behavsci-15-01623] Muis K. R., Duffy M. C. (2013). Epistemic climate and epistemic change: Instruction designed to change students’ beliefs and learning strategies and improve achievement. Journal of Educational Psychology.

[B37-behavsci-15-01623] Özmen H., Naseriazar A. (2018). Effect of simulations enhanced with conceptual change texts on university students’ understanding of chemical equilibrium. Journal of the Serbian Chemical Society.

[B38-behavsci-15-01623] Perry N. E., Brenner C. A., MacPherson N., Cleary T. (2015). Using teacher learning teams as a framework for bridging theory and practice in self-regulated learning. Self-regulated learning interventions with at-risk youth: Enhancing adaptability, performance, and well-being.

[B39-behavsci-15-01623] Perry N. E., Hutchinson L., Thauberger C. (2008). Talking about teaching self-regulated learning: Scaffolding student teachers’ development and use of practices that promote self-regulated learning. International Journal of Educational Research.

[B40-behavsci-15-01623] Pintrich P. R., Boekaerts M., Pintrich P. R., Zeidner M. (2000). The role of goal orientation in self-regulated learning. Handbook of self-regulation.

[B41-behavsci-15-01623] Richland L. E., Simms N. (2015). Analogy, higher order thinking, and education. Wiley Interdisciplinary Reviews: Cognitive Science.

[B42-behavsci-15-01623] Rosch E., Rosch E., Lloyd B. B. (1978). Principles of categorization. Cognition and categorization.

[B43-behavsci-15-01623] Sinatra G. M., Pintrich P. R. (2003). Intentional conceptual change.

[B44-behavsci-15-01623] Spruce R., Bol L. (2015). Teacher beliefs, knowledge, and practice of self-regulated learning. Metacognition and Learning.

[B45-behavsci-15-01623] Theobald M. (2021). Self-regulated learning training programs enhance university students’ academic performance, self-regulated learning strategies, and motivation: A meta-analysis. Contemporary Educational Psychology.

[B46-behavsci-15-01623] Tippett C. (2010). Refutation text in science education: A review of two decades of research. International Journal of Science and Mathematics Education.

[B47-behavsci-15-01623] Türk F., Çalik M. (2008). Using different conceptual change methods embedded within 5E model: A sample teaching of endothermic-exothermic reactions. Asia-Pacific forum on science learning and teaching.

[B48-behavsci-15-01623] Vamvakoussi X., Geary D. C., Berch D. B., Ochsendorf R. J., Mann Koepke K. (2019). The use of analogies in mathematics instruction: Affordances and challenges. Cognitive foundations for improving mathematical learning.

[B49-behavsci-15-01623] Vosniadou S. (2007). Conceptual change and education. Human Development.

[B50-behavsci-15-01623] Vosniadou S., Vosniadou S. (2013). Conceptual change in learning and instruction: The framework theory approach. International handbook of research on conceptual change.

[B51-behavsci-15-01623] Vosniadou S., Darmawan I., Lawson M. J., Van Deur P., Jeffries D., Wyra M. (2021). Beliefs about the self-regulation of learning predict cognitive and metacognitive strategies and academic performance in pre-service teachers. Metacognition and Learning.

[B52-behavsci-15-01623] Vosniadou S., Lawson M. J., Bodner E., Stephenson H., Jeffries D., Darmawan I. G. N. (2023). Using an extended ICAP-based coding guide as a framework for the analysis of classroom observations. Teaching and Teacher Education.

[B53-behavsci-15-01623] Vosniadou S., Lawson M. J., Gusti D., Wyra M., Van Deur P. (2019). Beliefs about learning and teaching questionnaire.

[B54-behavsci-15-01623] Vosniadou S., Lawson M. J., Wyra M., Van Deur P., Jeffries D., Ngurah D. I. G. (2020). Pre-service teachers’ beliefs about learning and teaching and about the self-regulation of learning: A conceptual change perspective. International Journal of Educational Research.

[B55-behavsci-15-01623] Vosniadou S., Skopeliti I. (2014). Conceptual change from the framework theory side of the fence. Science & Education.

[B56-behavsci-15-01623] Vosniadou S., Skopeliti I. (2019). Evaluating the effects of analogy enriched text on the learning of science: The importance of learning indexes. Journal of Research in Science Teaching.

[B57-behavsci-15-01623] Xu Z., Zhao Y., Zhang B., Liew J., Kogut A. (2023). A meta-analysis of the efficacy of self-regulated learning interventions on academic achievement in online and blended environments in K-12 and higher education. Behaviour & Information Technology.

[B58-behavsci-15-01623] Zimmerman B. J. (2002). Becoming a self-regulated learner: An overview. Theory Into Practice.

[B59-behavsci-15-01623] Zimmerman B. J. (2008). Investigating self-regulation and motivation: Historical background, methodological developments, and future prospects. American Educational Research Journal.

